# An AgNP-deposited commercial electrochemistry test strip as a platform for urea detection

**DOI:** 10.1038/s41598-020-66422-x

**Published:** 2020-06-12

**Authors:** Juanjuan Liu, Roozbeh Siavash Moakhar, Ayyappasamy Sudalaiyadum Perumal, Horia Nicolae Roman, Sara Mahshid, Sebastian Wachsmann-Hogiu

**Affiliations:** Department of Bioengineering, McGill University, Montreal, Quebec H3A 0C3 Canada

**Keywords:** Biomedical engineering, Sensors and biosensors, Materials for devices

## Abstract

We developed an inexpensive, portable platform for urea detection via electrochemistry by depositing silver nanoparticles (AgNPs) on a commercial glucose test strip. We modified this strip by first removing the enzymes from the surface, followed by electrodeposition of AgNPs on one channel (working electrode). The morphology of the modified test strip was characterized by Scanning Electron Microscopy (SEM), and its electrochemical performance was evaluated via Cyclic Voltammetry (CV) and Electrochemical Impedance Spectroscopy (EIS). We evaluated the performance of the device for urea detection via measurements of the dependency of peak currents vs the analyte concentration and from the relationship between the peak current and the square root of the scan rates. The observed linear range is 1–8 mM (corresponding to the physiological range of urea concentration in human blood), and the limit of detection (LOD) is 0.14 mM. The selectivity, reproducibility, reusability, and storage stability of the modified test strips are also reported. Additional tests were performed to validate the ability to measure urea in the presence of confounding factors such as spiked plasma and milk. The results demonstrate the potential of this simple and portable EC platform to be used in applications such as medical diagnosis and food safety.

## Introduction

Urea is an important biomarker for medical diagnosis^1^, which is a product of the urea cycle to lower down the toxic level induced by high concentration of nitrogen compound by converting ammonium ions into urea, and will be eventually eliminated by the kidney as urine^2^. As a result, the concentration of urea in serum can be used for disease diagnosis related to kidney and liver function^3^. In addition, the concentration of urea in food products such as milk is crucial for food safety^4^. For example, there have been reports of milk adulteration by adding urea into diluted milk to preserve the thickness and viscosity^5^. Therefore, accurate measurements of urea adulteration is important for health and food safety.

Biosensors convert the biological or chemical information into detectable signals with applications in medical diagnostics, food safety and environmental monitoring^6^. Based on different detection methods, there are various types of biosensors that have been developed for urea detection based on optical^7,8^, thermal^9^, piezoelectric^10^, or magnetic measurements^11,12^. In addition, Electrochemical (EC) biosensors, have attracted a lot of attention in urea detection recently, due to their simplicity and low limit of detection^13^.

EC biosensors directly convert the reaction of biological/chemical molecules to electrical response, providing a straightforward way to detect the target of interest^14,15^. A change in electrical current or potential is measured due to the reaction related to the analytes occurring on the electrode. In the case of voltammetry, the analyte is detected by measuring the corresponding current obtained by applying a varying potential between the working and reference electrodes. There are many different techniques based on the input modes of potential. Among them, CV is one of the most widely used^16^. For CV, potential is scanned between two fixed potential at a constant rate. Researches have been reported for biological detection via CV^17,18^.

Most biosensors for urea detection via electrochemical methods rely on enzymes such as urease to catalyze urea hydrolysis^19,20^. For example, urea can indirectly be detected by measuring the change in indicators such as photoluminescence (PL) intensity of quantum dots that are sensitive to the pH change induced by the reaction of urea or by measuring the change in the product under the catalysis of urease^3,21,22^. Au based sensors for urea detection have been explored mostly as enzymatic sensors, including Au electrode modified with other materials or electrodes such as ITO glass modified with AuNP. However, the utilization of an enzyme brings more complexity such as the immobilization of the enzyme which reduces its stability^23^. To address these issues, more non-enzymatic biosensors are being developed and have shown potential for urea detection^24–26^. More research examples on urea biosensors fabricated with nano-materials are discussed by Pundir *et al*. in a recent review article^27^.

Metallic nanoparticles draw significant attention in this field due to their chemical and physical properties compared to their bulk counterparts. When used in combination with EC electrodes, they exhibit several advantages such as high conductivity due to faster electron transfer, catalytic activity, and capturing affinity towards specific biomolecules^28^. These properties greatly facilitate the reaction of the analytes happening on metallic nanoparticles-modified electrode and increase the detection signal.

Ag-based biosensors have also been explored recently due to their good catalytic activity. Silver as a metal is not stable and it can be easily oxidized^29^. However, the oxidized compound of silver such as Ag(OH)_2_ acts as a catalyst that enables the hydrolysis of urea^23^. Ag coated zeolitic volconic tuff and ZnO nanorod structures have been reported for non-enzymatic Ag-biosensors operating in the range of µM to mM LOD^29^. Recently, a LOD in the range of nM (4.7 nM) has been reported using Ag-coated carbon nanotubes^23^.

There are many test strips for glucose detection commercially available made of plastic or paper substrates on which metallic layers (electrodes) are deposited for EC functionality^30^. Accu-chek aviva is one of them which is characterized by small physical dimensions, low cost, high accuracy and short measurement time for blood glucose estimation. The strip is composed of patterned Au-Pd electrodes (channels) deposited on a plastic substrate. In this article, we report the modification of this glucose EC strip with AgNPs for the detection of urea.

This work is aiming for a platform that is flexible, portable, and inexpensive for the detection of urea via electrochemistry. For this purpose, it is important to choose materials that are highly conductive and catalytically active. As mentioned above, there are already commercial strips available for glucose detection via EC. Naturally, to decrease the cost for materials, the use of commercial strips with good conductivity and low cost is a reasonable option. Moreover, since AgNPs have been shown to exhibit catalytic reactivity towards urea detection^23,29^, we built a novel device that uses AgNPs on an inexpensive and readily available EC test strip. Therefore, in this article, the development and characterization of this novel EC substrate based on a commercial glucose test strip, on which AgNPs were deposited, were reported. All biochemical components like enzymes on the surface of the electrode were first removed to make sure that the channels are only coated with Au and Pd as conductive materials. The morphology of the substrate and the distribution of AgNPs were characterized by SEM and optical microscopy. Further, urea was used as a test molecule in our set-up due to the demand for accurate measurements in biomedical applications where urea levels are important. Furthermore, we tested our device at mM concentrations, since this is the normal physiological range of urea in blood.

The sensing system uses three different channels on the commercial strip, which were selected as the 3-electrode system and connected to a potentiostat. The utilization of the whole strip makes this sensor a portable and simple platform that avoids the use of complex EC cells typically seen in a regular 3-electrode system.

## Results and discussion

**Figure 1:**
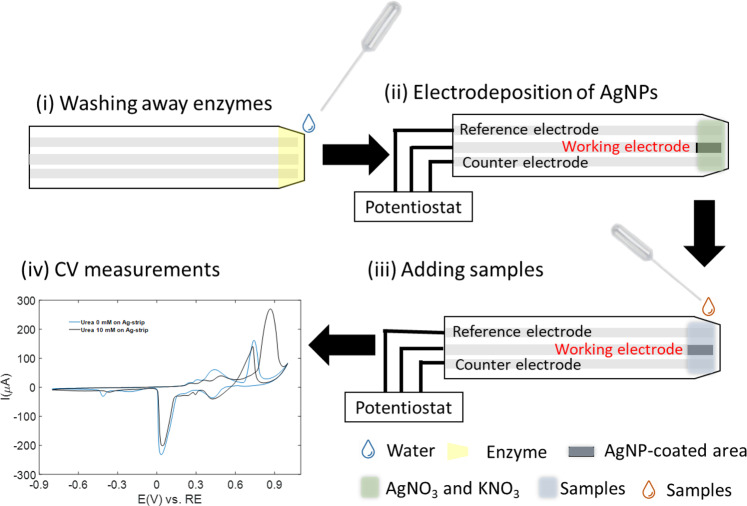
Schematic representation of the workflow. (i) preparation of the test strip before modification (by removing the enzyme layer). (ii) electrodeposition of AgNPs directly on the test strip by applying a potential at −0.6 V for 30 s between the working and reference electrodes in presence of AgNO_3_ and KNO_3_. (iii) drop casting of the sample on the sensing area of the test strip for detection. (iv) EC measurements.

The representation of the steps followed for the preparation of the sensor and sample is presented in Fig. 1. To build the system, we first removed all the components such as the enzymes for glucose test with ethanol and then distilled water (Fig. 1(i)). The removal is evaluated visually by observing the disappearance of the yellow color associated with the enzyme (Fig. S1). It is followed by the electrodeposition of AgNPs on the working electrode in the sensing area by applying a potential at −0.6 V for 30 s (Fig. 1(ii)) between the working and reference electrodes in the presence of AgNO_3_ and KNO_3_. The samples were then drop-cast onto the sensing area (Fig. 1(iii)) for measurements and performance characterization. (Fig. 1(iv)).

### Choice of materials

For this work, we chose a substrate that is easy to fabricate, consisting of AgNPs and a commercial glucose test strip. To achieve good performance of the biosensor substrate for EC detection, the conductivity and catalytic activity of the electrode are important. The glucose test strip we used here is composed of a plastic substrate deposited with Au and Pd bimetallic channels. Compared to the commonly used electrodes such as Au, glassy carbon electrode (GCE), and screen-printed electrodes (SPE), this substrate is inexpensive and easy to obtain. Moreover, it consists of several channels that can work as a typical 3-electrode electrochemical system, which confers portability to this complex EC system.

However, urea cannot be detected by the test strip alone without any modification. As a result, in addition to the test strip, other material(s) that provide catalytic activity the test strip for urea detection is (are) necessary. Research has shown that Ag after oxidation can catalyze the hydrolysis of urea. In addition, nanoparticles provide a larger surface area compared to bulk materials. Therefore, AgNPs are selected to functionalize the substrate for urea detection and for simplicity are grown *in situ* via electrodeposition.

### Fabrication and characterizations of AgNP-coated electrode

**Figure 2:**
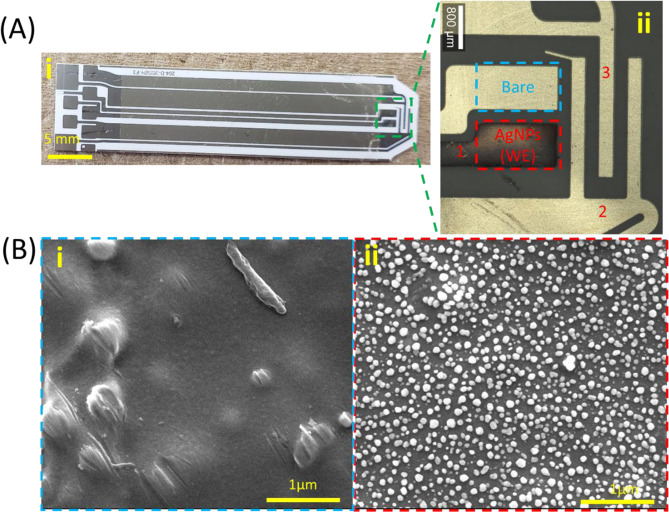
The fabrication and characterization of the test strip. (**A**) the photo (i) and optical imaging (ii) of commercial glucose test strip before (i) and after (ii) electrodeposition of AgNPs (inset: 1-working electrode; 2-counter electrode; 3-reference electrode); (**B**) SEM images of bare test strip (i) and AgNP-coated test strip (ii).

To prepare the AgNP-coated test strip, the commercial Au strip was peeled off (Fig. 2A(i)) and AgNPs were then coated on the surface of this electrode by electrodeposition. After the electrodeposition of AgNPs on the electrode, a dark layer on the surface is observed due to the formation of AgNPs (Fig. 2A(ii)). To further confirm the deposition and observe the morphology of AgNPs, microscopy images and SEM images of both bare electrode (test strip) and AgNPs-coated electrode were recorded (Fig. 2B(i) vs. (ii)). The result showed that the bare electrode is relatively flat. The deposited AgNPs add distinct features to the electrode and are relatively uniform sized at 30–100 nm. Several conditions of electrodeposition regarding the constant potential and time applied were adjusted to get better deposition of AgNPs. A potential of −0.6 V for 30 s was finally decided to be used due to a better uniformity of the nanoparticle layer.

**Figure 3:**
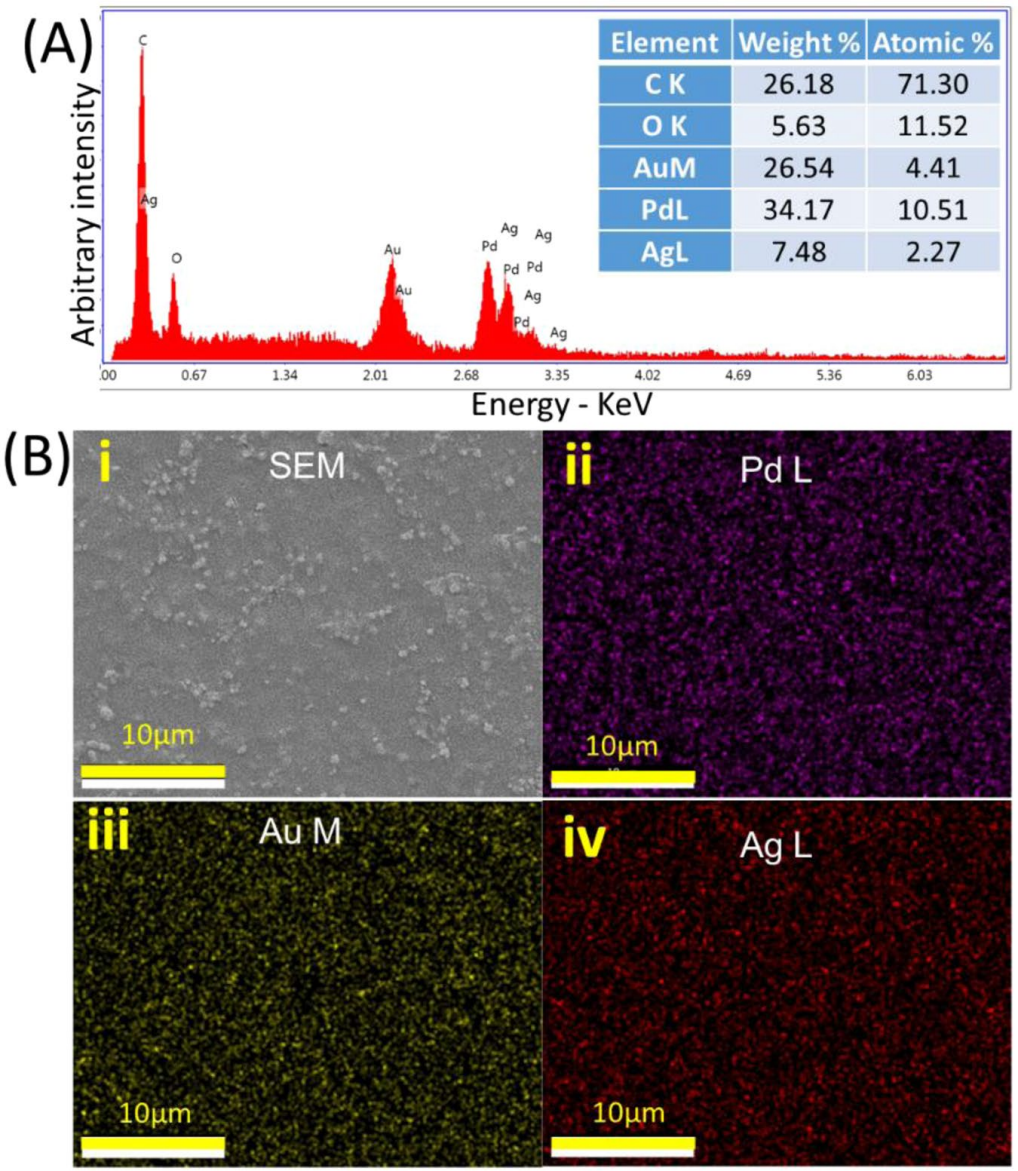
EDS characterization of the test strip. (**A**) EDS spectrum and (**B**) EDS mapping of the AgNP-coated commercial glucose test strip.

To further confirm the deposition of AgNPs on the surface, EDS was conducted on AgNP-coated electrodes (Fig. 3). The results showed that the bare electrode is composed of both Au and Pd. Ag was also detected on the substrate after the deposition (Fig. 3A). The distribution of each elemental composition was also demonstrated with EDS mapping that confirms the successful coating of AgNPs (Fig. 3B).

### Electrochemical evaluation of the electrode

**Figure 4:**
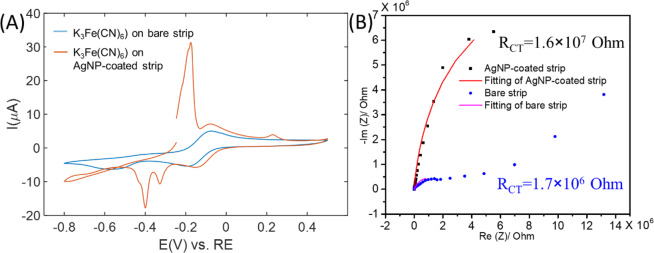
EC evaluation of the AgNP-coated test strip. (**A**) Electrochemical evaluation with K_3_Fe(CN)_6_ and (**B**) EIS of bare and AgNP-coated glucose test strip. Electrolyte: 0.1 M KCl for K_3_Fe(CN)_6_ (5 mM), and 0.1 M NaOH for EIS; scan rate for CV: 20 mV/s.

Ferricyanide (K_3_Fe(CN)_6_) is a typical test molecule used to characterize electrochemical performance of an electrode. To confirm the electroactivity of the electrodes before and after the modification with AgNPs, 5 mM potassium ferricyanide in 0.1 M KCl was added. AgNP-coated electrode showed similar oxidation and reduction peaks of ferricyanide and ferrocyanide conversion, with slightly higher peak current and peak potential, indicating a comparable electrochemical reactivity and conductivity of the modified electrode (Fig. 4A). It is important to note that, compared to the bare electrode, the modified electrode with AgNPs exhibited an extra peak during forward scanning, in addition to the oxidation peak from ferricyanide. This is due to the fact that Ag can be easily oxidized. In addition, EIS was performed to characterize the change in impedance of the electrode upon the deposition of AgNPs (Fig. 4B), which was indicated by the resistance of charge transfer R_CT_. The results show that after the deposition of AgNPs, the impedance increases by one magnitude (from 10^6^ Ohm for bare strip to 10^7^ Ohm for AgNP-coated test strip). This is likely due to the fact that the AgNPs deposited on the electrode are not continuously distributed (as shown in Fig. 2B(ii)). Another possible reason is that the formation of the oxidation layer of Ag such as AgO and Ag_2_O decreases the electrical conductivity of the strip, which may result in a higher impedance^31^.

### Urea detection

**Figure 5:**
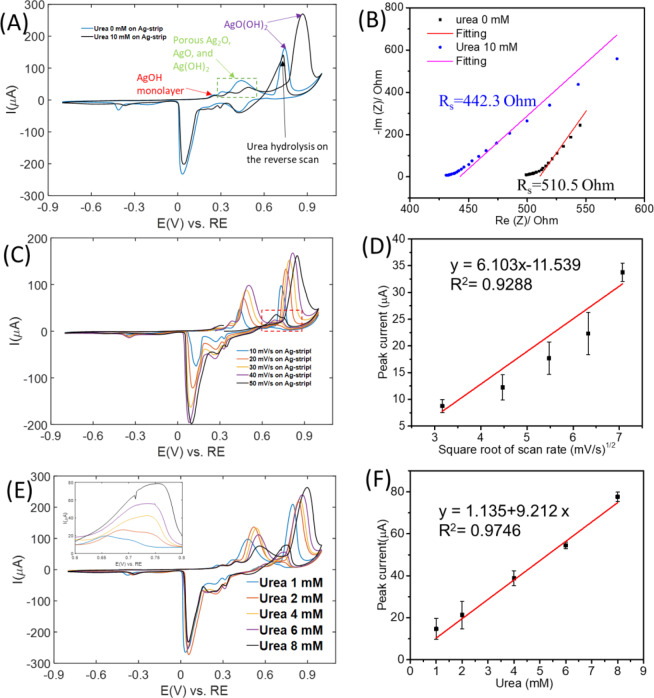
Sensing performance of the AgNP-coated test strip for urea detection. (**A**) CV response and (**B**) EIS of AgNP-coated glucose test strip in the absence and presence of urea; (**C**) CV response of AgNP-coated glucose test strip in the presence of urea at different scan rates (10–50 mV/s), and (**D**) corresponding calibration plot of peak current vs. square root of scan rates. (**E**) CV response of AgNP-coated glucose test strip in the presence of urea at different concentrations (1–8 mM) and (**F**) corresponding calibration plot of peak current vs. urea concentration.

The electrodes are then characterized for their catalytic activity as related to the detection of urea. CV and EIS were performed on the AgNP coated strip electrode in 0.1 M NaOH in the absence and presence of urea (Fig. 5A,B). The effective area of the electrode considered as working electrode is ~2.5 mm^2^ (Fig. 2A(ii)). It is demonstrated that for same range of applied potential (−0.8–1 V), the current response in the absence and presence of urea are quite different (Fig. 5A,B). The change in the impedance of the electrolyte (solution impedance R_s_) after adding urea is presented in Fig. 5B. It shows that the addition of urea results in a decrease of the impedance of the electrolyte, which corresponds to the increase in the current observed in the CV curves. In addition, it has been reported and mentioned that the electrodeposition of AgNPs provides high selectivity towards urea hydrolysis^23,29^. Thus it is necessary to make sure that Au and Pd on the original substrates are not interfering the detection of urea. To confirm that, control experiments to test the performance of the bare strip for urea detection were conducted, as well as to confirm the catalytic function of AgNPs. CV responses were also recorded on bare test strip in the absence and presence of urea (Fig. S2). Since there are no peaks related to urea hydrolysis in these curves, we can conclude that the bare strip is not able to detect urea without the catalytic contribution from AgNPs.

In the presence of urea, the AgNP-coated electrode, shows multiple peaks on the forward scanning. This is due to the oxidation of AgNPs on the surface, which leads to the formation of different layers on the surface of the electrode during the forward scanning, such as, Ag_2_O, AgO, and Ag(OH)_2_ (Fig. 5A)^29,32^. On the reverse scanning, a peak near 0.7 V appears, due to the oxidation of urea under the catalysis of Ag(OH)_2,_ (also observed in other reports)^23^, shown in Fig. 5A. This peak demonstrates that the AgNP-coated electrode has catalytic activity for the detection of urea in alkaline electrolytes due to the formation of Ag(OH)_2_. The peak observed in forward scanning near 0.85 V corresponds to the reaction of the catalyst Ag(OH)_2_, while the peak near 0.7 V on the reverse scanning corresponds to the hydrolys is of urea under the catalysis of Ag(OH)_2_ (Eqs. 1 and 2). The overall electrocatalytic urea oxidation reaction catalyzed by the oxidation product of Ag can be summarized in Eqs. 1–4. As a result, the electrodeposition of AgNPs on the electrode improves the ability to detect urea due to higher catalytic reactivity.

Oxidation:

1 $${\rm{Ag}}{({\rm{OH}})}_{2}+2{{\rm{OH}}}^{-1}\to {\rm{AgO}}{({\rm{OH}})}_{2}+{{\rm{H}}}_{2}{\rm{O}}+2{{\rm{e}}}^{-1}$$

2 $${\rm{AgO}}{({\rm{OH}})}_{2}+{{\rm{H}}}_{2}{\rm{O}}+{\rm{CO}}{({{\rm{NH}}}_{2})}_{2}\to {\rm{Ag}}{({\rm{OH}})}_{2}+{{\rm{CO}}}_{2}+{{\rm{N}}}_{2}$$

Reduction:

3 $$2{{\rm{H}}}_{2}{\rm{O}}+2{{\rm{e}}}^{-1}\to {{\rm{H}}}_{2}+2{{\rm{OH}}}^{-1}$$

Overall:

4 $${\rm{CO}}{({{\rm{NH}}}_{2})}_{2}+{{\rm{H}}}_{2}{\rm{O}}\to {{\rm{CO}}}_{2}+{{\rm{N}}}_{2}+{{\rm{H}}}_{2}$$

After morphological, elemental, and electrochemical characterization of the electrode, we investigated its sensing performance by measuring the dependence of redox peaks on the square root of scan rates and the concentration of urea. The normal range of urea in blood is 2.5~7.5 mM^12^. Therefore, in our measurements we used concentrations of urea in the range of 1–8 mM, such that it better mimics the concentrations in real blood samples.

The effects of scan rates on the peak potential and the peak current were studied by measuring CV curves at scan rates ranging from 50 mV/s to 10 mV/s with the modified electrode in 0.1 M NaOH containing 2 mM urea (Fig. 5C,D). A linear relation between the peak current and the square root of the scan rate was observed (Fig. 5D), with a correlation coefficient 0.9288, indicating that the redox process is diffusion-controlled. Based on the Randles-Sevcik equation^33^, the diffusion coefficient of urea is then calculated to be 2.5 × 10^−3^ cm^2^/s.

To further evaluate the sensitivity of the modified electrode, the current dependence of the urea oxidation peak on the concentration was investigated (Fig. 5E,F) by recording the CV response at a series of concentrations. A linear relationship between the concentration and the peak current was observed with a correlation coefficient 0.9746. The resulting LOD is 0.1419 mM and is comparable with value reported in the literature for measurements with other EC sensors (Table 1), while at the same time we utilize a simpler sensor that was built by modifying inexpensive commercial glucose EC strips.

**Table 1:** Comparison of electrochemical electrodes reported in the literature for urea detection.

Type	Materials and morphology	Technique	LOD	Sensitivity	Linear detection range	Ref.
Enzymatic	Self-assembled monolayer of dihydroxythiophenol modified gold electrode with urease	LSV	0.2 mM	—	0.2–5 mM	^35^
	Amine functionalized hyperbranched AuNP modified ITO glass electrode modified with urease	CV	10 μM	7.48 nA/mM	10 μM-35 mM	^36^
	Polyaniline-Nafion/Au/ceramic composite	CV	0.5 μM	3.162 mAmM^−1^cm^−2^	10–100 μM	^22^
	Glassy carbon electrode was electrochemically modified with Fe3O4/MWCNT/PANI-Nafion nanocomposite film with bacterial enzyme	CV, DPV, CA	67 µM	—	1.0–25.0 mM	^37^
	Urease-immobilized graphene nanoplatelets and graphitized nanodiamonds.	Direct current voltage (IV)	83.3 µM	48.1 µAmM^−1^cm^−2^	—	^38^
Non-enzymatic	Gold Electrodes Modified with Peptide Self-Assemblies 4-mercaptopyridine (MCP) and L,L-diphenylalanine micro/nanostructures (FF-MNSs) (Benzene rings and amide groups interacts with NH_4_^+^)	CV	0.17 mM	2.83 μAmM^−1^cm^−2^	0.1–1 mM	^39^
	AgNP-decorated nitrogen doped single wall carbon nanotubes	CV	4.7 nM	141 μAmM^−1^cm^−2^	66 nM – 20.6 mM	^23^
	Graphite composite electrode based on natural zeolitic volcanic tuff modified with silver	SWV	50 μM	0.058 mA/mM	0.2–1.4 mM	^29^
	Sputtered Ag on zinc oxide (ZnO) nanorod-structures grown on a carbon paper substrate	CA	13.98 μM	0.1622 μAμM^−1^cm^−2^	26.3–450 μM	^40^
	Glassy carbon modified with nickel sulfide/graphene oxide	CV, DPV	3.79 μM	—	10–50 μM	^41^
	Au electrode deposited with Ni	CV	33.5 μM	52.20 μAmM^−1^cm^−2^	—	^42^
	NiO nanosheet	CA	2 μM	3.4 A/(M cm^2^)	4.4–181.6 μM	^43^
Our work, non-enzymatic	AgNP-deposited commercial Au-Pd electrode	CV	141.9 μM	9.212 μA/mM	1–8 mM	

### Reproducibility, reusability, stability, and selectivity of the EC strip

**Figure 6:**
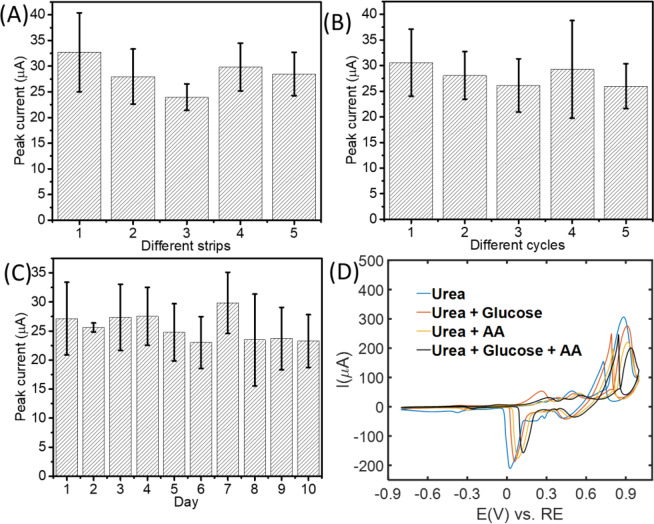
CV response of AgNP-coated glucose test strips in the presence of urea. (**A**) with different strips, (**B**) different cycles with the same strip, (**C**) with strips stored for different times, and (**D**) with interference of other substances glucose and AA. Electrolyte: 0.1 M NaOH; Scan rate: 50 mV/s.

The reproducibility of different strips and the reusability regarding multiple cycles with one strip were investigated via CV responses (3 consecutive cycles for reproducibility) with different strips and different cycles. For reproducibility evaluation, 5 strips here were tested in 10 mM urea solution in NaOH electrolyte (Fig. 6A). The relative standard deviation (RSD) of the peak current for these 5 different strips was 7.33% after 3 consecutive cycles, which indicates that the detection of urea using different electrodes/strips is reproducible.

The reusability of the AgNP-coated strip was also investigated by performing multiple cycles on the same electrode consistently (n = 4) (Fig. 6B) in 2 mM urea solution in NaOH electrolyte. The result showed that the oxidation peak changes over time. However, since the test strips we fabricated here are inexpensive (<$2/strip), they are disposable, and their reusability might not be a concern.

The stability of the strips for storage was tested for 10 days to evaluate the shelf lifetime. AgNP-coated strips were prepared via electrodeposition of AgNPs on the first day. They were then stored under vacuum to prevent the oxidation of Ag. 2~3 strips were used for urea detection every day for 10 days in a row to see if the strips after 10 days storage can still function for urea detection via EC. On the other hand, because the oxidation of Ag is necessary prior to the oxidation of urea, for practical purposes it should be acceptable that the test strips are stored in air (rather than under vacuum). The peak currents of urea oxidation were recorded and plotted versus time (different days) (Fig. 6C). The results indicate that after 10 days, the fabricated strip is still functional to urea detection even the variability appears. This result shows that our strips have potential for commercialization.

Since glucose is a common component in human blood and ascorbic acid (AA) plays a vital role in metabolic processes, the selectivity of AgNP-coated strip electrode in the presence of AA and glucose was evaluated by performing CV in urea in the presence of these two analytes. As shown in Fig. 6D, in the presence of urea, after the addition of glucose and/or AA, the oxidation peak of urea was shifted, and the peak current also changed. However, there were no other peaks corresponding to the interference of the additional analytes. This indicates that urea can still be detected on the AgNP-coated strip in the presence of glucose and AA.

### Urea detection in reconstituted plasma solution and milk samples

To investigate the potential of the substrate for applications in medical diagnosis and food safety, the modified test strips were used for the detection of urea in urea-spiked solutions of plasma and milk. Different concentrations of plasma and milk samples were prepared. CV responses were collected (Fig. S3), and the recovery rates were calculated and presented in Table 2. Since we are aiming to achieve urea detection in samples that are close to the original concentration of the potential sample with less dilution and preparation, only two lowest dilutions (highest detectable ratios) were shown here (1:10 and 1:5).

**Table 2:** Detection of urea in milk and plasma sample.

Ratio of milk in NaOH electrolyte (n = 5)	1:10	1:5
Urea added (mM)	10	10
Total found (mM)	8.937581	3.14036
RSD %	13.9	14.0
Recovery %	89.38	31.40
Ratio of plasma in NaOH electrolyte(n = 5)	1:10	1:5
Urea added (mM)	10	10
Total found (mM)	3.108446	1.201476
RSD %	26.4	26.86
Recovery %	31.1	12.0

To perform urea determination in treated milk sample, CV responses and the peak currents corresponding to urea oxidation for different concentrations of milk samples in NaOH electrolyte (100 mM) were collected. Based on the calibration plot, the detected concentration of urea was calculated and compared to the actual spiked concentration. The recovery rates were then calculated. The results show that to detect urea with a higher recovery rate in real milk sample, it is important to dilute the milk sample to a proper ratio. In this set-up, we observed that urea detection is more accurate for a higher dilution of milk sample in NaOH electrolyte.

In addition, the detection of urea in plasma sample was performed following a similar procedure as described for the detection in milk. CV responses and peak currents were collected with different concentrations of plasma sample in electrolyte buffer. Compared to the detection of urea in milk sample, the detection of urea with similar sample dilution ratio is less accurate due to the interference from the more complicated components in plasma. As a result, our AgNP-decorated electrode is more suitable for urea detection in milk samples. However, it is also important to note that our results in plasma and milk samples indicate that the platform will need to be further improved before it can be used in practical applications. This can be achieved by improvements to the sample preparation method that can lead to a better recovery rate and optimization of the catalytic material for improved sensitivity and reproducibility.

## Conclusions

Urea detection is important for diagnosis and food safety applications. Significant research, both related to methods and materials, is dedicated towards the development of biosensors that can provide valuable data for these applications. In this work, a flexible electrochemical sensor device for urea detection was developed by decorating the working electrode of a commercial glucose test strip with AgNPs. This provides an inexpensive and portable platform that leverages existing technology and describes improvements for the specific use case described here. The commercial strip was first modified by removing the enzyme layer and then by electrodepositing AgNPs on the surface of the working electrode. The uniform deposition of AgNPs improves the surface area and leads to better performance for electrochemical measurements. Furthermore, the presence of AgNPs on the surface of the working electrode and the interaction with the electrolyte acts as a catalyst in the hydrolysis of urea and allows for its detection. The sensing performance of the strip was studied by CV measurements. The linear dependence of the peak current on the square root of scan rates indicates a diffusion-controlled electrochemical process. On the other hand, the linear detection range between 1–8 mM shows the potential of this platform for real application in diagnosis. Moreover, the reproducibility, reusability, storage stability, and selectivity of this substrate were evaluated to validate its potential for practical applications. The platform was also used for urea detection in samples that mimic real environments such as plasma and milk. Although more improvements are still needed to be further used in for urea detection in real samples, the results still show promising potential of the substrate for urea detection within the physiological concentration range in human blood as well as in milk for possible contamination. It is also worth noticing that more improvements in reproducibility and selectivity need to be made in order to make this platform useful for point of need applications.

## Materials and methods

### Materials

Silver nitrate, potassium nitrate, sodium hydroxide, plasma and urea were purchased from Sigma-Aldrich. They were used as received without any treatment. The commercial glucose test strip (Accu-chek aviva glucose test strip) was purchased from a local pharmacy. Milk was purchased from a local shop.

### Preparation of AgNP-coated test strip

The enzyme coating the original strip (which is used for glucose measurement) was chemically removed by washing with ethanol and distilled water, and the plastic coverage on the electrode end was physically peeled off. Next, a AgNPs coating was created by electrodeposition as described in^34^. Briefly, it was conducted with a typical 3-electrode system in an EC cell with a reference electrode, a counter electrode and a working electrode. The EC set-up was built directly on the whole test strip with three channels selected as the three electrodes (working electrode, counter electrode, and reference electrode) (Fig. 2A). For the fabrication of AgNP-coated test strip, electrodeposition was performed. Briefly, 30 µL of a mixed solution including silver nitrate (AgNO_3_) and potassium nitrate (KNO_3_) was dropped on the tip of the strip substrate. A constant potential −0.6 V (vs. reference electrode) was applied for 30 s. This value was confirmed by exploring the reduction potential via CV in AgNO_3._ It could be observed that after the electrodeposition a darker layer of AgNPs was formed on the surface of the test strip (Fig. 2A(ii)).

### Scanning electron microscopy (SEM)

SEM images of the electrode with/without the deposition AgNPs were obtained using a Carl Zeiss SEM instrument at high vacuum with the acceleration voltage of 5 kV equipped with Quanta 450 FE-SEM that can be used to obtain Energy Dispersive Spectroscopy (EDS) mapping. These images provide information regarding the morphology of the surface condition of the test strip electrode and the deposition status of the AgNPs.

### Electrochemical measurement

All the EC measurements are performed with a single-channel Potentiostat (BioLogic Science Instrument, SP-150, France) controlled by the EC-lab software installed on a PC. The EC system was set up by connecting the three electrodes to a Potentiostat device. The electrolyte used for urea detection was NaOH (0.1 M, 30 µl in the whole strip system). To avoid fluctuations in the signal, the second or third cycles (if not specified) of CV curves after stabilization of the first scan were recorded as the reaction occurred at the working electrode. The currents were examined and shown as the voltammograms. The limit of the detection was then calculated by the following relation: LOD = 3 SD/S, where LOD is the limit of detection, SD is the standard deviation of blank measurement when there is no urea, and S is the slope of the linear equation.

### Urea detection in milk

To remove the excess fat and proteins from the purchased milk, it was pretreated by mixing 5 ml of milk with 10 ml acetonitrile. The mixture was then centrifuged at 10,000 rpm for 3 min. After centrifugation, the supernatant was collected as the ready milk sample for urea detection. The electrolyte NaOH at 0.1 M was prepared with the milk sample at different ratios, e.g., treated milk sample was diluted different times in the electrolyte. 10 mM of urea was then prepared in milk sample diluted in NaOH (1:100, 1:50, 1:10, 1:5, 1:1). For each concentration of milk sample, 5 measurements on different substrates were collected.

### Urea detection in plasma

The plasma was firstly 10 times diluted in phosphate buffered solution (1X, pH 7.4) before using it. Similar to the preparation of milk sample, the diluted plasma was then diluted with NaOH electrolyte at different ratios/concentrations (1:100, 1:50, 1:10, 1:5, and 1:1). 10 mM of urea was then prepared in these samples. 5 measurements were collected with different strips for each condition.

## Supplementary information

Supplementary information.
