# Supplementary material for: An AgNP-deposited commercial electrochemistry test strip as a platform for urea detection
Source: Sci Rep. 2020 Jun 12;10:9527. doi: 10.1038/s41598-020-66422-x (PMC7293235; doi:10.1038/s41598-020-66422-x)
Supplement: Supplementary file 1 — Supplementary information. [file 41598_2020_66422_MOESM1_ESM.docx]

**Supporting information**

**An AgNP-deposited commercial electrochemistry test strip as a platform for urea detection**

**Juanjuan Liu^1^, Roozbeh Siavash Moakhar^1^, Ayyappasamy Sudalaiyadum Perumal^1^, Horia Nicolae Roman^1^, Sara Mahshid^1^, Sebastian Wachsmann-Hogiu^1,*^**

^1^Department of Bioengineering, McGill University, Montreal, Quebec, H3A 0C3, Canada

*** Correspondence:**
[Sebastian.wachsmannhogiu@mcgill.ca](mailto:Sebastian.wachsmannhogiu@mcgill.ca)


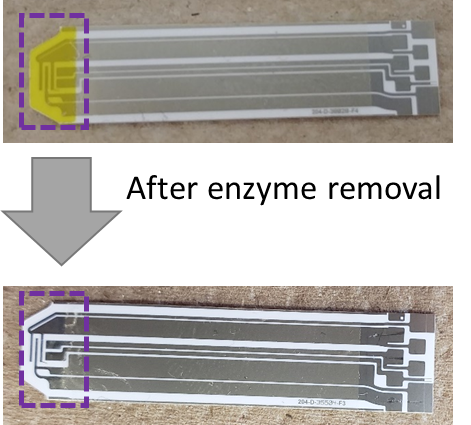


**Figure S1**. Evaluation of enzyme removal from the test strip. The yellow color of the enzyme (indicated in the upper figure, purple rectangle) disappears after washing with ethanol and distilled water (lower figure, purple rectangle).


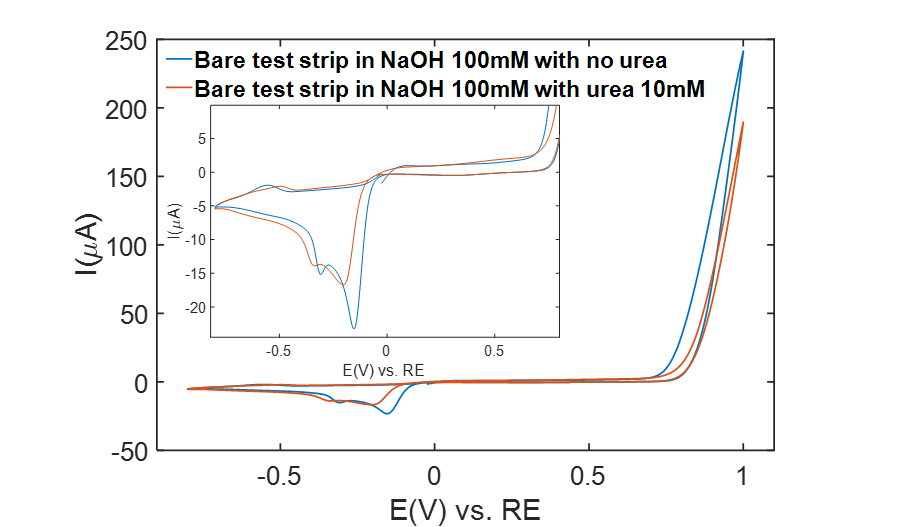


**Figure S2**. CV response of the bare test strip (without AgNP deposition) in the absence and presence of urea. Electrolyte: NaOH 0.1M, scan rate: 20 mV/s


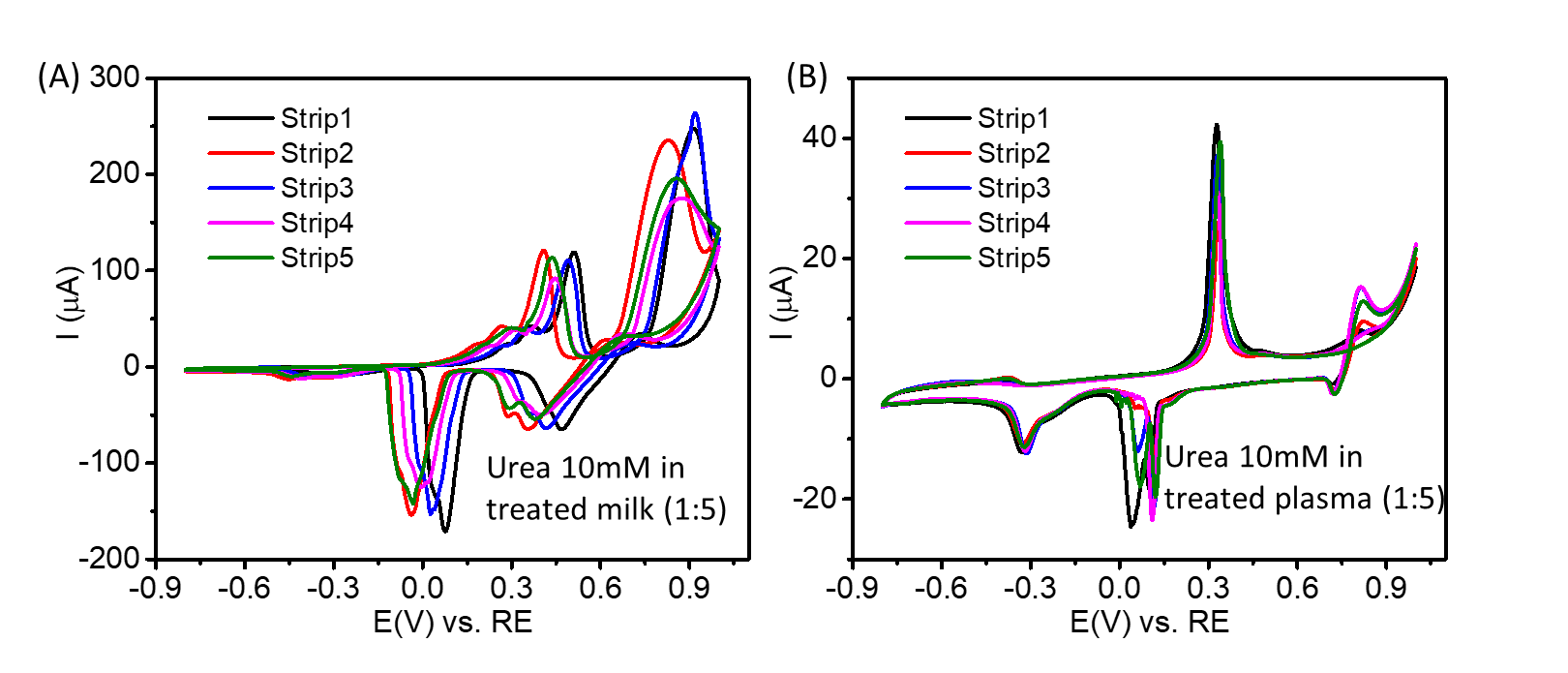


**Figure S3**. CV response of AgNP-coated glucose test strips for the detection of urea in milk (A) and plasma (B) samples
